# Recent Advances in Barrier Property Enhancement of Biodegradable Polymer Films: Strategies and Challenges

**DOI:** 10.3390/nano16150934

**Published:** 2026-07-29

**Authors:** Cheng Huang, Shuangshuang Yue, Lei Zhong, Sheng Huang, Min Xiao, Shuanjin Wang, Dongmei Han, Yuezhong Meng

**Affiliations:** 1 School of Chemical Engineering and Technology, Sun Yat-sen University, Zhuhai 519082, China; huangch255@mail2.sysu.edu.cn (C.H.);; 2 Key Laboratory of Low-Carbon Chemistry & Energy Conservation of Guangdong Province, State Key Laboratory of Optoelectronic Materials and Technologies, School of Materials Science and Engineering, Sun Yat-sen University, Guangzhou 510275, China; yueshsh3@mail.sysu.edu.cn (S.Y.);; 3 Institute of Chemistry, Henan Academy of Sciences, Zhengzhou 450052, China

**Keywords:** biodegradable polymers, packaging materials, oxygen/water barrier properties

## Abstract

Over the past decades, biodegradable polymer films have gained significant attention owing to their environmental friendliness and abundant raw material availability. However, the barrier properties of biodegradable polymers as packaging materials remain inferior to those of traditional petroleum-based plastic films, limiting their suitability for applications sensitive to oxygen or moisture such as food preservation, pharmaceutical packaging, and electronic device protection. In this paper, we summarize effective strategies to enhance the barrier properties of biodegradable polymer packaging films, such as surface coating, polymer blending, multilayer compounding, copolymerization and modification, nanocomposite technology, and filler reinforcement. Subsequently, we discuss the application of these strategies to biodegradable polymers, including both natural and synthetic varieties. A central focus is elucidating the relationship between the modification approaches, molecular structure, and oxygen/water barrier properties in high-barrier biodegradable polymer materials, aiming to provide valuable guidance for the development of advanced biodegradable polymer packaging materials. Finally, we present the existing challenges and future prospects for the development of high-barrier biodegradable polymer packaging materials.

## 1. Introduction

Since the 1950s, the global consumption of polymer materials has surged dramatically due to the convenience and high-end value in the packaging field. Annual production has soared from 234 million tons in 2000 to 430 million tons in 2023, with a sustained annual growth rate of 5.2% [1,2,3]. It is well recognized that a variety of commercial items—such as food, pharmaceutical products, and electronic equipment—are prone to degradation caused by oxygen and water vapor. For packaging materials, two critical parameters that dictate the shelf life of commodities are the oxygen transmission rate (OTR) and water vapor transmission rate (WVTR). Protecting these goods from damage, from production to consumer use, has always been a challenge for packaging materials. It is estimated that the proportion of fresh food lost before it reaches the store is as high as 20–25% due to inadequate packaging, and in developing countries, the figure can even reach 50% [4,5]. The active components present in pharmaceutical products exhibit high sensitivity to oxygen and water vapor. Ineffective packaging not only diminishes the therapeutic efficacy, but also leads to deterioration of pharmaceuticals, thereby creating a potential risk to human health [6]. Furthermore, electronic components, such as printed circuit boards (PCBs), crystal diodes, and sensors, are also exceedingly vulnerable to the effects of water vapor and oxygen. Effective packaging functions as a barrier layer, which not only safeguards the stability and dependability of electronic devices, but also prolongs the operational lifespan of these products [7,8]. Therefore, effective and sensible packaging can improve both the functionality and value of the items being packaged. It can protect goods from the influence of the external environment during storage, transportation, reprocessing and sales, thereby preserving their quality and safety.

While polymer packaging such as plastics brings convenience and high value to processes such as storage, transportation, reprocessing and distribution, the environmental pollution associated with it has attracted widespread global concern. According to reports, more than 340 million tons of plastic waste are produced globally each year, of which about 46% comes from the packaging industry [9]. While the recycling rate of plastics has risen from 0 in the 1980s to the current estimate of 19.5%, packaging materials, particularly those used for food, remain largely non-recyclable. At present, roughly 95% of packaging materials crafted from polyolefins and polyethylene terephthalate (PET) fail to undergo recycling. After being used just once for a brief period, these materials are sent directly to landfills, an outcome that causes an annual global economic loss ranging from $80 to $120 billion. Thus, substituting petroleum-derived polymers with more sustainable alternatives is an urgent global issue that requires resolution [10]. Biodegradable polymer packaging materials offer an effective solution to these issues, as these materials can decompose into water and carbon dioxide (CO_2_) under natural environments within approximately six months, whereas non-degradable plastics, such as polyolefins, can persist for several centuries.

Despite extensive scholarly efforts to advance the use of biodegradable polymers in packaging applications, few such polymers available on the market satisfy the high-barrier requirements of modern food packaging as demanded by society. This discrepancy arises from the inferior oxygen/water vapor barrier properties of biodegradable polymers when compared to traditional petroleum-based plastics. Therefore, enhancing the oxygen and water vapor barrier capabilities of biodegradable polymers is critical, as is exploring the interconnections between the barrier properties of distinct biodegradable polymers and their molecular structures.

Biodegradable polymers, such as cellulose, starch, protein, chitosan, polylactic acid (PLA), poly(propylene carbonate) (PPC), poly(butylene adipate terephthalate) (PBAT), and polycaprolactone (PCL), among others [1], exhibit notable advantages regarding raw material sources and environmental friendliness. However, the market application of biodegradable materials has been limited due to poor processability, such as insufficient mechanical and thermomechanical performance, or due to their barrier properties against oxygen and water vapor failing to meet the strict requirements of commercial high-barrier packaging materials. In order to fulfill the strict barrier standards required for packaging commodities like food, pharmaceuticals, and electronic devices, it is necessary to enhance the barrier properties of biodegradable polymers against oxygen, water vapor, and odor compounds. For example, the oxygen permeability (OP) for instant coffee packaging should be maintained below 5 cm^3^/(m^2^·day). Pharmaceuticals and electronic components require materials with even lower rates of OP and water vapor permeability (WVP). For instance, the materials used to protect organic light-emitting diode (OLED) devices must have an OTR that does not exceed 10^−3^ cm^3^/(m^2^·day); the WVTR must not exceed 10^−6^ g/(m^2^·day) [11,12].

To improve the barrier properties of biodegradable polymer packaging materials, researchers have developed numerous innovative technologies [13]. These technologies can generally be classified into five categories: surface coating, multilayer compositing, blending, copolymerization and modification, and nanocomposites and nanotechnology. Numerous techniques and corresponding materials have been applied for packaging applications across food, pharmaceuticals, electrical devices, daily necessities, and cosmetics.

This review introduces the fundamental principles of barrier materials and summarizes the primary approaches to enhancing the barrier properties of packaging materials. The types of high-barrier biodegradable polymers and their recent progress in enhancing barrier function are highlighted. By reviewing various methods for enhancing the barrier properties of biodegradable polymers, this paper summarizes the advantages, disadvantages, efficacy, and commonality of different strategies, insights that offer potential reference value for the future development of biodegradable polymers.

## 2. Barrier Properties of Packaging Materials

**Figure 1:**
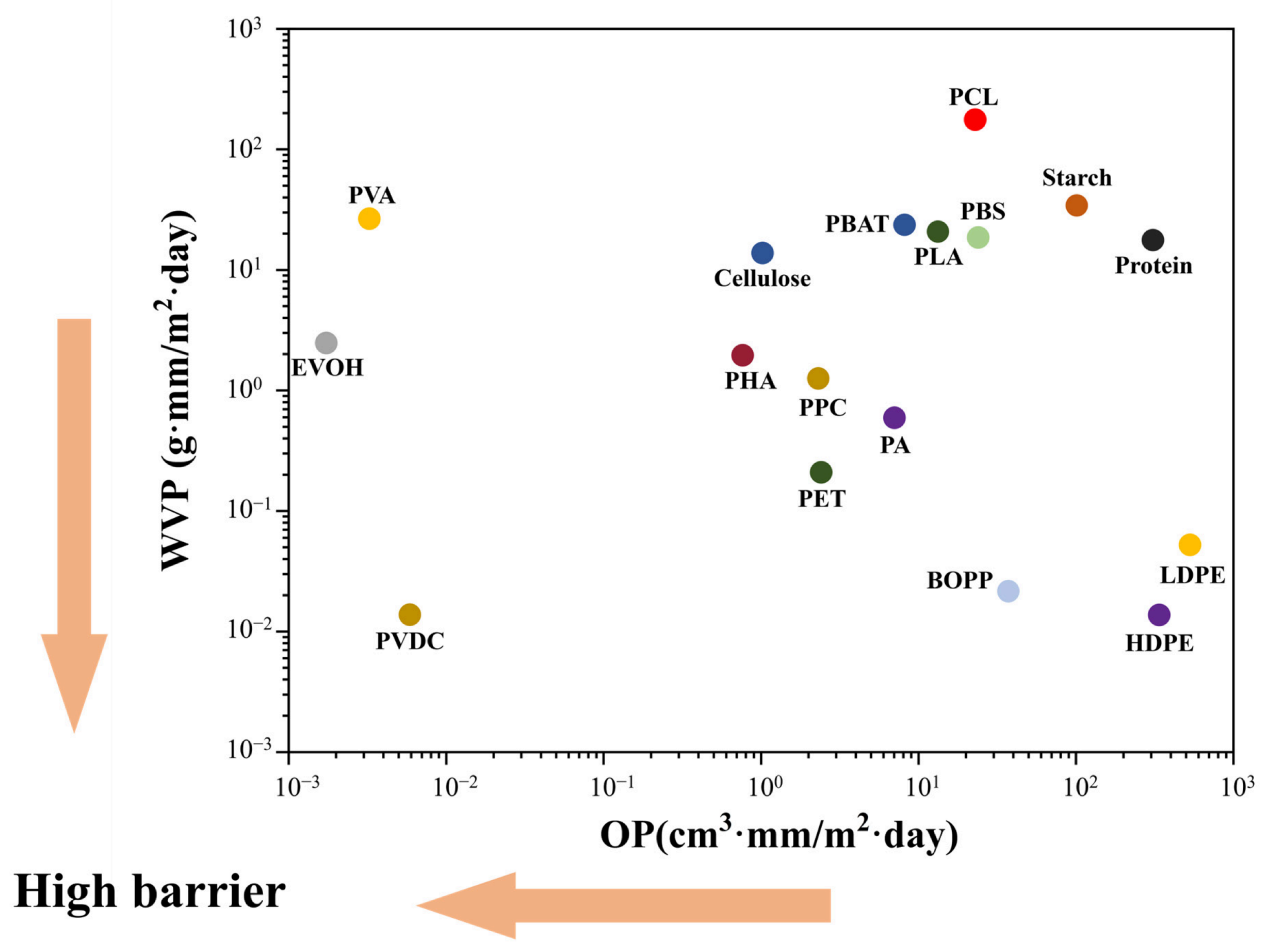
OP and WVP of frequently used polymer packaging materials and biodegradable polymers.

In order to guarantee that food, medicine, electronic devices and other products do not deteriorate or sustain damage during transportation and utilization, it is imperative that packaging materials exhibit superior gas barrier, mechanical and thermal performance. Specifically, the gas barrier properties of the materials include water vapor and oxygen barrier properties, which directly influence the shelf life and service life of the goods. Consequently, optimizing the water vapor and oxygen barrier properties of packaging materials is essential to preserve the quality of products. Figure 1 illustrates the OP and WVP of frequently used polymer packaging materials and biodegradable polymers, including polymers such as PET, biaxially oriented polypropylene (BOPP), high-density polyethylene (HDPE), low-density polyethylene (LDPE), polyamide (PA), polyvinylidene chloride (PVDC), and ethylene vinyl alcohol copolymer (EVOH) [14,15,16,17], and biodegradable polymers such as cellulose, starch, protein, chitosan, PLA, polybutylene succinate (PBS), PBAT, PPC, polyhydroxyalkanoate (PHA), PCL, and polyvinyl alcohol (PVA) [18,19,20,21,22,23,24,25,26,27,28,29,30,31,32].

### 2.1. Barrier Principles

#### 2.1.1. Principles of Permeation

**Figure 2:**
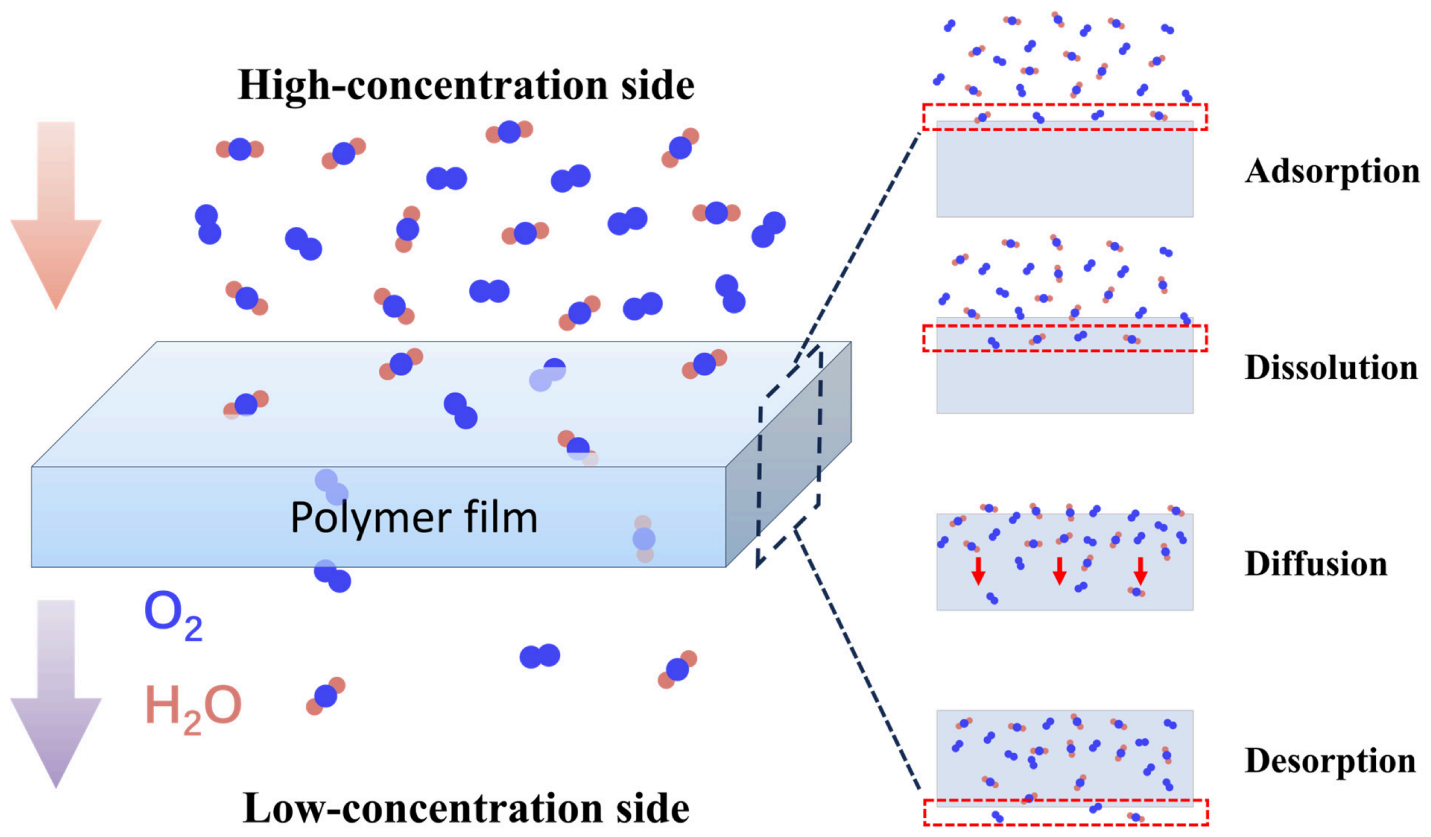
Permeation process of gas molecules in polymer films [35]. Blue and orange dots represent O_2_ and H_2_O molecules, respectively; red dashed boxes highlight the four permeation stages (adsorption, dissolution, diffusion, desorption); arrows indicate the direction of molecular transport.

Barrier properties serve a pivotal function in packaging materials. High-barrier materials are defined as polymer materials that can significantly obstruct and slow down the permeation of small gas molecules, substances widely utilized in the storage and transportation of commodities such as foods, pharmaceuticals, and electronic products. To further improve the barrier properties of materials, conducting in-depth research into the basic principles of gas molecule permeation through packaging films is a necessary first step [33,34]. The structural diversity of polymer units and intrinsic voids in the molecular chains create permeation pathways in the polymer matrix, making it challenging to completely prevent the penetration of small gas molecules. Figure 2 illustrates the permeation process of gas molecules through polymer films. This process primarily involves four steps:

(1) Gas molecules are adsorbed onto the polymer film surface.
(2) Gas molecules gradually dissolve in the polymer film.
(3) Gas molecules diffuse through the polymer film interior with a definitive concentration gradient.
(4) Gas molecules are desorbed and detach from the opposing surface of the polymer film.

The adsorption and desorption of gas molecules on the surface of the material occur at a relatively rapid rate. Therefore, the penetration rate is primarily determined by the dissolution and diffusion rates [36]. The dissolution and diffusion rates of gas molecules within polymer membranes largely depend on the structure of the membrane, especially with regard to the free volume fraction, rigidity and flexibility of the molecular chain, the connection mode of structural units, substituent groups, and crystallinity of the polymer [37,38,39]. For instance, polypropylene (PP) and polyethylene (PE) only have non-polar carbon–hydrogen chain structures, with extremely low surface energy and strong hydrophobicity. Their molecular chains are simple and regular, and easy to crystallize, but the amorphous regions sandwiched between crystalline domains exhibit looser chain arrangement and lower local molecular packing density compared with crystalline regions. As a result, PP and PE exhibit good water resistance but inadequate oxygen barrier properties. PVDC contains numerous polar functional groups on its molecular chain and a high degree of regularity in the molecular chain structure. The volume effect and dipolar interactions of the chlorine atoms make the molecular chains abnormally tightly aligned and have very high crystallinity. This high crystallinity and tight packing greatly diminish the free volume between molecular chains and restrict the mobility of molecular chains. Therefore, PVDC has superior oxygen and water vapor barrier properties. However, the exceptionally high crystallinity and packing density make PVDC lacking in processing characteristics.

#### 2.1.2. Different Test Methods for Oxygen and Water Vapor Permeability

The barrier properties of polymer packaging materials can be characterized using OTR and WVTR, or OP and WVP. The OTR and WVTR are defined as the volume or weight of oxygen or water vapor that passes through a film per unit area and time at equilibrium under specific test conditions (temperature, humidity). The transmission rate of the gas molecules varies with the thickness and type of the film material. The OP and WVP are the volume or weight of gas molecules passing through the film per unit volume and time at equilibrium. They are thus commonly used to characterize the permeability of films and are independent of the test area and film thickness. In addition, the permeability of the same sample may vary depending on the test conditions, as different standards specify different test temperature and humidities.

##### Oxygen Permeability

OP assessment methods are primarily classified as isobaric and differential pressure techniques based on different testing principles. In the isobaric method, the test sample is secured in the permeation chamber of the apparatus, which is separated into two compartments. Oxygen and nitrogen, respectively, are introduced on opposite sides of the test chamber, with equal total pressure but different oxygen partial pressures. Under the influence of an oxygen concentration gradient, oxygen passes through the test sample and enters the coulometric sensor along with the nitrogen, where it undergoes a chemical reaction to produce a voltage. This voltage bears a direct proportional relationship to the quantity of oxygen that passes through the coulometric sensor per unit time. The test is completed when a steady-state oxygen concentration is achieved in the nitrogen compartment. The OTR cm^3^/(m^2^·day) is calculated from the experimental results. The OP cm^3^·mm/(m^2^·day) is calculated using Equation (1).

OP = OTR × *d* (1)

where *d* represents the thickness of the test specimen in millimeters (mm).

In the differential pressure method, the sample sealed in the test chamber partitions the chamber into two separate compartments. First, both the low-pressure and the high-pressure chamber are evacuated. Then, a test gas is introduced into the evacuated high-pressure chamber and permeates through the sample into the low-pressure chamber. The permeability of the gas is calculated from the change in gas concentration in the low pressure chamber.

##### Water Vapor Permeability

Testing for water vapor barrier properties primarily includes weighing and sensor methods. The principle of the weighing method is to fill a cup with a measured quantity of water in a room with a certain temperature and humidity, and then cover the cup with the sample to be tested. The sample is secured to the rim of the cup using a silicone gasket and metal screws. In order to minimize the impact of temperature and humidity conditions on the initial weight of the sample, the sample is preconditioned under constant temperature and humidity for 24 h. The WVTR g/(m^2^·day) is calculated from the change in weight over the 24 h. The weighing method provides the benefits of operational simplicity and cost-effectiveness. The WVP g·mm/(m^2^·day·bar) is calculated using Equation (2):

WVP = (WVTR × *d*)/Δ*p* (2)

Δ*p* represents the difference in partial water vapor pressure between the two chambers of the test sample (bar), and *d* represents the thickness of the film (mm).

Based on the sensor used, the sensor method can be classified into electrolytic sensor technology, infrared sensor technology, or humidity sensor technology. The sample under test is mounted in a test chamber that is divided into an upper dry chamber and a lower humid chamber. The pressure in both test chambers is identical, with dry nitrogen on one side and nitrogen at a specific humidity on the other side. Owing to the existence of a humidity gradient, water vapor permeates from the humid chamber through the sample into the dry chamber and is conveyed to an infrared detector, which generates a quantifiable electrical signal. Once the test reaches a steady state, the WVTR g/(m^2^·day) of the samples is calculated from the electrical signal. The WVP g·mm/(m^2^·day) is calculated using Equation (3):

WVP = WVTR × *d* (3)

where *d* represents the thickness of the film (mm).

### 2.2. Methods to Improve Barrier Properties

The application prospects of some polymer materials in high-barrier packaging are limited by their inadequate oxygen and water vapor barrier properties, such as PBAT and PP. Consequently, researchers have centered their efforts on developing approaches to improve the barrier properties of biodegradable polymers. These methods include surface coatings, multilayer composites, blending, copolymerization and modification, and nanocomposites and nanotechnology [1,40].

#### 2.2.1. Surface Coating

Typically, single-component polymer films have low intrinsic barrier properties. Surface coating technology has been developed to overcome this limitation. This technology uses physical or chemical techniques to apply a dense, highly impermeable layer on the polymer substrate. The permeability of the overall composite material is significantly decreased by several orders of magnitude owing to this coating arrangement. As a result, coating-treated polymer substrates have emerged as the best options for safeguarding delicate goods like food, medications, and organic electronic components. The commercialization of this technology follows fifty years of development. The most popular coating types on polymer substrates, including high-barrier inorganic or organic materials like silicon oxide (SiO_x_), PVDC, and PVA/montmorillonite (MMT), using various techniques, will be compiled in the following section in order to improve their barrier properties. Physical vapor deposition (PVD), chemical vapor deposition (CVD), dip coating, and layer-by-layer self-assembly (LBL) are examples of coating processes [38,41,42,43].

##### Inorganic Surface Coating

PVD and plasma chemical vapor deposition (PCVD) are widely employed to deposit nanoscale inorganic coatings on substrates such as PET, BOPP, and other polymeric materials. In the PCVD technique, metals or their oxides are melted and vaporized under high-vacuum conditions, and then cooled and deposited onto a substrate film to form a high-barrier coating [44]. For instance, aluminum or SiO_x_ is deposited on substrates such as PET, PP, or PLA using the PCVD method to form a dense and uniform barrier layer [45,46].

Ohishi et al. reported a method to form dense SiO_x_ coatings with excellent gas barrier properties by applying polysilazane coatings to PET and polyimide (PI) substrates, followed by curing under low-temperature conditions [47,48]. This method has the flexibility of the liquid-phase coating process, but can also realize the transformation at a lower temperature, avoiding the damage to the polymer substrate. The most common precursor solution for SiO_x_ is fully hydrogenated polysilazane (PHPS), which has a basic chemical structure of [-Si(H_2_)-NH-], and can be hydrolyzed and pyrolyzed to activate the photolytic oxidation reactions of the Si-N, N-H and Si-H bond to form SiO_x_ compounds. Furthermore, the transformation process is a weight-gaining reaction, and the degree of coating densification becomes higher. Consequently, the PHPS conversion method holds significant application value in the fabrication of gas barrier coatings for polymer films. Several researchers have investigated the transformation of PHPS under ultraviolet (UV) irradiation, and the gas barrier properties of the composites were markedly enhanced when the coated PHPS coatings were cured by UV irradiation. Morlier et al. [49] reported that the conversion of the PHPS coatings to SiO_x_N_y_ under UV irradiation resulted in excellent barrier properties, with an OTR of 0.06 cm^3^/(m^2^·day) and a WVTR of 0.2 g/(m^2^·day).

##### Organic Surface Coating

Organic surface coating is a technique that involves applying organic materials such as polyacrylic acid (PAA), PVA, or PVDC to a polymer substrate through specialized techniques to form a protective coating. This method is characterized by a relatively simple industrial production process, broad applicability, and the ability to significantly enhance the barrier properties of polymer materials.

Both PAA and PVA possess excellent oxygen barrier characteristics, while PVDC simultaneously exhibits outstanding barrier properties against both oxygen and water vapor. However, the poor thermal processability of these materials limits their direct application, leading to their common use as coatings on polymer substrates. A prime example is BOPP, where such coatings significantly enhance the gas barrier properties of the base material. BOPP films are fabricated by mechanically stretching PP films in both the transverse and longitudinal directions at high temperatures. BOPP demonstrates excellent transparency, tensile strength, chemical resistance, and dimensional stability, making it widely applicable in packaging for food, floral, apparel, and labeling applications. However, BOPP films are prone to surface and internal defects during the stretching process, which affects their gas barrier properties and prevents them from being used in packaging materials that require high standards of barrier properties. To enhance the barrier properties of BOPP, Daniloski et al. fabricated acrylic (Ac)/PVDC-coated BOPP/Ac/PVDC films. Testing revealed that the OTR of the BOPP/Ac/PVDC composite films (0.98~324 cm^3^/(m^2^·day·bar)) was notably lower than that of the pure BOPP films (227~6200 cm^3^/(m^2^·day·bar)) [50]. Lu et al. prepared PET/MDI composite films by coating PET films with 4,4′-diphenylmethane diisocyanate (MDI), PAA, and borax, and the oxygen barrier properties of the films were significantly enhanced. The OTR decreased from 75 cm^3^/(m^2^·day) to less than 0.005 cm^3^/(m^2^·day), a value approaching the permeability of EVOH film, the current leading material in oxygen barrier properties [41].

##### Inorganic/Organic Surface Coating

Organic/inorganic materials can be used as hybrid nano-coatings fabricated by LBL and impregnation flow-induced assembly methods to further enhance the gas barrier properties of the substrate materials.

The LBL assembly technique has emerged as a leading approach for constructing organic/inorganic hybrid nano-coatings [42]. This method involves the sequential and alternating deposition of oppositely charged polyelectrolytes onto a substrate, forming a precisely controlled multilayer structure. Due to its exceptional tunability in film thickness and composition, LBL assembly is widely employed in applications including sensors, biomedicine, energy storage, water purification, and gas barrier packaging. Typical materials used in LBL fabrication include polyelectrolytes such as PAA, poly(vinyl phosphonic acid) (PVPA) and poly(sodium 4-styrenesulfonate) (PSS), combined with nanosheet fillers such as MMT, vermiculite (VMT) and graphene oxide (GO). A critical factor in optimizing barrier properties is the orientation of these nanofillers—they must be aligned perpendicular to the diffusion path of permeating molecules to maximize resistance. The LBL technique excels in controlling nanofiller alignment by leveraging electrostatic interactions: charged nanofillers are attracted to oppositely charged polymer surfaces, ensuring their flat deposition and optimal perpendicular orientation relative to molecular diffusion.

Despite its many benefits, the LBL technique faces limitations in large-scale production. Recently, researchers proposed a method for fabricating organic/inorganic hybrid nano-coatings as an alternative to LBL: a simple dip-coating process combined with a flow-induced orientation method. This approach involves immersing a polymer film in a low-viscosity aqueous dispersion containing inorganic nanosheets and a polymer binder, followed by vertical suspension and oven drying. As the dispersion flows downward during drying, it aligns the nanosheets perpendicular to the film’s thickness direction, creating an ordered multilayer structure of alternating polymer and inorganic layers.

The coating’s composition and thickness can be precisely tuned by adjusting the impregnation solution’s concentration and the polymer-to-inorganic ratio, enabling control over both structure and barrier properties. This method offers distinct advantages over conventional LBL assembly, particularly in terms of scalability for continuous industrial production. For instance, Zhang et al. successfully implemented this flow-induced co-assembly technique to deposit transparent PVA/MMT nano-coatings on PET substrates. The films produced in this way displayed significantly enhanced oxygen barrier properties while maintaining excellent optical clarity [43].

#### 2.2.2. Multilayer Composite

Combining two or more polymers into multilayer composites offers an effective approach for developing advanced polymeric materials. This strategy leverages synergistic interactions between components to enhance material performance [51,52]. Currently, the fabrication methods for polymer multilayer films can be divided into multilayer dry lamination and multilayer melt coextrusion processes.

Multilayer Dry Lamination Method: This method utilizes a variety of films or sheets as substrates that are first coated with an adhesive layer, followed by drying or curing before being laminated with other film types to improve material performance. For instance, high-performance composite films are prepared by combining PTFE and aluminized polymer substrates with polyurethane through the dry lamination method. These composite films exhibit improved gas barrier properties along with enhanced mechanical strength [53,54].

Multilayer Coextrusion: Multilayer coextrusion simultaneously processes two or more distinct polymers into an integrated layered structure. By combining materials with complementary characteristics, this technique produces composite films exhibiting enhanced performance, including enhanced flexibility, gas barrier properties, mechanical strength and aesthetics. The multilayer coextrusion method is extensively employed in manufacturing packaging films, pipes, containers, and other applications and can provide properties beyond those of a single material.

Multilayer coextrusion often faces interfacial compatibility challenges when combining dissimilar polymers. To address this, adhesive layers are typically incorporated between incompatible materials to improve interlayer bonding. Plastic products manufactured using the multilayer coextrusion technique exhibit excellent barrier properties and mechanical strength. LDPE/adhesive layer/EVOH/adhesive layer/LDPE is a representative multilayer coextrusion material, extensively utilized in the packaging of cosmetics. The barrier properties of composite films improve with the number of layers, so the barrier properties of the film can be regulated through the lamination process [55]. There are two general types of multilayer coextrusion techniques for synthesizing composite films with 2 to 11 layers or thousands of layers. The first process, in which two or more distinct polymer types are combined and assembled into coextruded structures of 2 to 11 layers, has been widely adopted by industry. However, this method has some drawbacks in that the number of layers that can be extruded is limited by the number of extruders available [56]. The second method utilizes a multiplier to separate the melt stream from the feed blocks. Such systems are capable of producing multilayer films ranging from tens to thousands of layers, where the final number of layers is determined by the number of multipliers used. However, when the number of layers reaches thousands, the material is limited to two different polymers [57].

#### 2.2.3. Blending

Polymer blending is an effective processing technique that combines multiple polymers to develop materials with improved performance characteristics. This method integrates the unique advantages of different polymers to compensate for the limitations of a single material. The resulting blends exhibit optimized barrier properties, mechanical strength, thermal properties, chemical stability and transparency for specific application requirements. For instance, blending a high-oxygen-barrier material with a high-water-vapor-barrier material results in a blended polymer that has both low OP and low WVP. Common blending systems include blends of PP or PET with PA, and blends of PP, PE or PA with EVOH [51,58].

Polymer blending is typically performed under high shear forces, such as in an extruder or internal mixer, facilitating improved dispersion and enhanced compatibility between different polymers. However, when certain polymers exhibit poor compatibility during blending, compatibilizers are required to enhance interfacial interactions and reduce interphase voids and defects, ultimately enhancing the blended material’s gas barrier and mechanical properties. The technique of polymer blending is extensively applied in automotive parts, electronic device housings, packaging materials and medical devices.

#### 2.2.4. Copolymerization and Modification

Polymer copolymerization and modification represent an effective strategy for enhancing the performance of materials through tailored copolymer synthesis. This technique involves chemically bonding two or more distinct monomers to create customized polymer chains with precisely controlled characteristics. By strategically selecting monomers with specific functional properties, researchers can precisely engineer copolymers to achieve desired physical, chemical, or mechanical properties for specific applications. Modification of copolymers can be categorized into four types:

(1) Random copolymerization, where monomers are combined in a random manner to form uniformly distributed copolymer chains.
(2) Block copolymerization, where different types of monomers are polymerized into block structures.
(3) Graft copolymerization, where different side chains are attached to a main chain. The side chains consist of one monomer type, whereas the main chain is made up of a different type.
(4) Alternating copolymerization, where two types of monomers are arranged in an alternating sequence, forming a regular structural pattern.

Different copolymerization methods can enhance different properties of the material, including barrier properties, thermal stability, mechanical strength, chemical resistance and processability. Some modified copolymers may also use two or more modification methods.

#### 2.2.5. Nanocomposites and Nanotechnology

**Figure 3:**
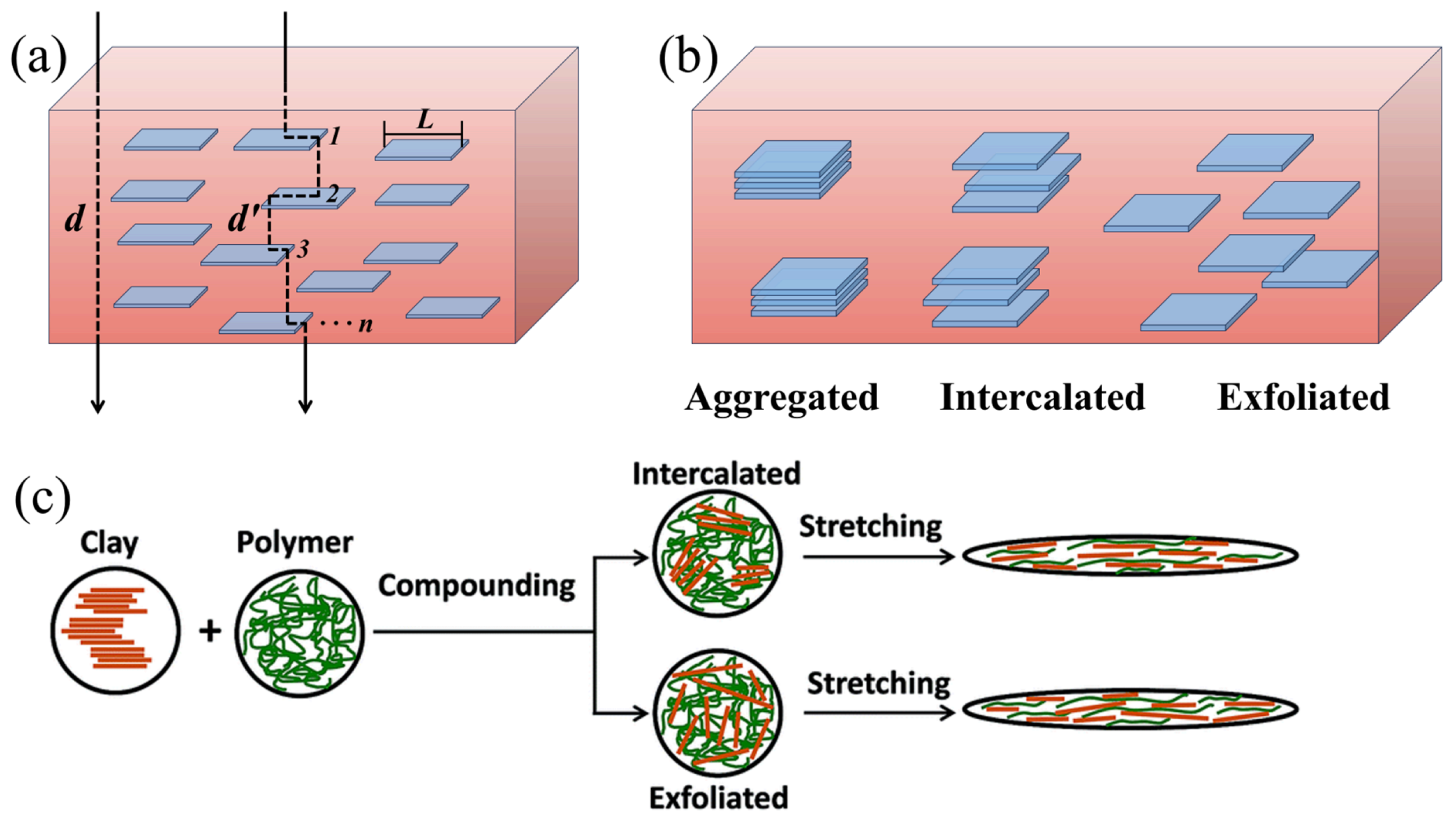
(**a**) Schematic diagram of gas molecules passing through a polymer matrix filled with inorganic nanosheets; *d* represents the film thickness, *d*′ represents the actual diffusion path length, *L* represents the length of nanosheets, and numbers 1, 2, 3, …, *n* indicate the number of nanofillers bypassed by gas molecules. (**b**) schematic representation of the three morphologies of inorganic nanosheets in composite materials; (**c**) schematic of the stretch–exfoliation–orientation process [61].

In recent years, significant progress has been made in the research on nanosheet-based polymer nanocomposite films as high-performance barrier materials. Studies have demonstrated that the homogeneous dispersion of nanosheets within polymer matrices can remarkably improve the gas barrier properties of composite films. The underlying mechanism primarily involves two aspects: firstly, the nanosheets generate a “tortuous path” effect in the polymer matrix, forcing gas molecules to follow extended diffusion pathways; secondly, the nanosheets themselves function as “nano-barrier walls” that effectively impede gas molecule permeation. This unique barrier mechanism enables the composite films to achieve permeability reductions of several orders of magnitude. In 1967, Nielsen pioneered a groundbreaking geometrical model to describe gas permeation through nanocomposite membranes [59]. The filler in a composite membrane can be roughly divided into particles or flakes of length *L* and thickness *W*. The particles and flakes are assumed to be impermeable to gas molecules, with no strong interactions between the gas molecules and either the polymer film or the filler. Consequently, gas molecules must diffuse around these nanofillers, thereby extending the diffusion distance from the membrane thickness *d* to *d*′ [60]. As shown in Figure 3a, if a gas molecule diffuses from the top to the bottom of a membrane, it must bypass *n* nanoparticles or sheets. The distance the gas molecule must bypass is calculated using Equation (4):

*d*′ = *d* + *nL*/2 (4)

The diffusion process of gas molecules within a film is primarily determined by the diffusion path. Thus, the relative permeability coefficient of a composite membrane decreases with increasing diffusion path length.

Polymer matrix materials frequently employed in nanocomposite applications include PE, PA, PP, and PET. Inorganic nanofillers primarily comprise metals and their oxides, various clays, and graphene [48]. According to the dispersion state of inorganic nanofillers, nanocomposites are categorized into three types, aggregated, intercalated, and exfoliated, as shown in Figure 3b. In the aggregated structure, the inorganic nanofillers are uniformly distributed in the polymer matrix, while maintaining a multilayer structure. In the intercalated structure, the polymer chains penetrate into the interlayer space of the inorganic nanofillers, leading to expansion of the interlayer distance. In the exfoliated structure, the inorganic nanolayers are fully separated and uniformly dispersed in the matrix as individual layers. The exfoliated structure represents the most desirable configuration as it endows composites with outstanding gas barrier properties, thermal stability, and mechanical strength at low nanofiller loadings [62].

To improve the exfoliation degree and dispersion of inorganic nanofillers in the polymer matrix, thereby maximizing nanocomposite properties, it is generally necessary to conduct physical or chemical modifications on both the fillers and the substrate. These modifications aim to facilitate exfoliation during the compounding process and improve polymer compatibility. For instance, polystyrene (PS)/(MMT) nanocomposite films can be produced via a combined biaxial stretching and extrusion method. As shown in Figure 3c, the mechanical stretching process enhanced the exfoliation and orientation of MMT within the PS matrix, which effectively improved the barrier properties of PS/MMT composite films. When the MMT content in the PS matrix reached 2.7 vol%, the OTR and WVTR reduced by 30% and 50%, respectively, while the optical performance decreased only slightly [61].

At present, surface coating, multilayer lamination, polymer blending, copolymer modification, and nanocomposites and technologies remain the most promising methods for the preparation of high-barrier polymer packaging materials [1,13]. Surface coating is a well-established and widely used high-barrier technology that chemically or physically deposits a high-barrier coating on a polymer substrate to improve the storage and transportation life of various goods [63]. Polymer blending is a straightforward method to fabricate high-barrier polymer packaging materials, where polymers with superior barrier properties act as the dispersed phase in an affordable substrate with inferior barrier properties to prepare composite materials [64]. Multilayer compounding involves combining two or more polymers to form new polymer materials. This approach is particularly useful when phase separation occurs during the blending of different polymer components. Multilayer composite methods enable more efficient utilization of the characteristics of each component, thereby enhancing the mechanical and barrier properties of the composite. Copolymerization modification is a method of improving the properties of materials by synthesizing copolymers, in which two or more different monomers are chemically reacted to form a copolymer chain. The selection of specific monomers can be directed to improve the physical, chemical or mechanical performance of the copolymer to meet the requirements of a particular application. Nanocomposites and nanotechnology are a dynamic and innovative area of research. Researchers have shown interest in applying these materials to fabricate high-barrier membranes, a focus that can be attributed to the “tortuous path effect” or the “nano-barrier effect” presented by the highly impermeable nanoplates [65,66]. It is noteworthy that novel nanoplatelets such as graphene and its derivatives, hexagonal boron nitride (hBN), layered double hydroxides (LDH), and MXene show promising potential for developing high-barrier polymer encapsulation materials.

While these methods can partially address the barrier property deficiencies in single-component polymer films, significant challenges remain to be overcome. These challenges include the brittleness and poor interfacial compatibility of surface coatings, the choice of polymer blend morphology, and the large-scale production of nanocomposites with polymers.

## 3. Biodegradable Polymer Packaging Materials

The focus on polymer films as packaging materials has increased significantly in recent decades, making them highly competitive alternatives to conventional packaging materials like glass, wood, metal and ceramics. This is mainly due to their light weight, functional designability, low manufacturing costs and ease of processing. While these plastic-based materials offer unparalleled convenience and functional benefits, their persistent environmental impact has emerged as a global sustainability challenge, prompting urgent calls for eco-friendly alternatives and improved waste management strategies [2]. On the one hand, their production and processing constitute an energy-intensive life cycle stage, generating substantial greenhouse gas emissions including CO_2_, CH_4_, and NO_x_ that directly drive global climate change. On the other hand, the combustion of plastic waste releases hazardous byproducts, notably carbon monoxide (CO), hydrogen chloride (HCl), diolefins, dioxins, and acetaldehyde, which present dual threats: acute environmental contamination through atmospheric pollution and soil infiltration, and chronic health risks via bioaccumulation in ecosystems and human populations [67,68].

In recent years, with the implementation of “plastic restriction order” and “plastic prohibition order” policies, the steady rise in public environmental awareness, and the resource depletion stemming from the overuse of traditional petroleum-based polymers, several countries have proposed the prohibition of single-use and non-biodegradable plastic products. Biodegradable plastics, which can be decomposed by microorganisms into water, CO_2_ and environmentally benign organic matter, have thus attracted significant research attention in the packaging materials field. Although biodegradable materials like PLA, PBAT, and starch-based polymers have made certain progress as alternatives, they still exhibit limitations in gas barrier properties. To tackle this critical limitation, a comprehensive understanding of the interrelationship between the oxygen/water vapor barrier properties of various biodegradable polymers and their material composition/structure is important, and this may help improve their barrier properties and facilitate their commercialization.

### 3.1. Classification of Biodegradable Polymers

**Figure 4:**
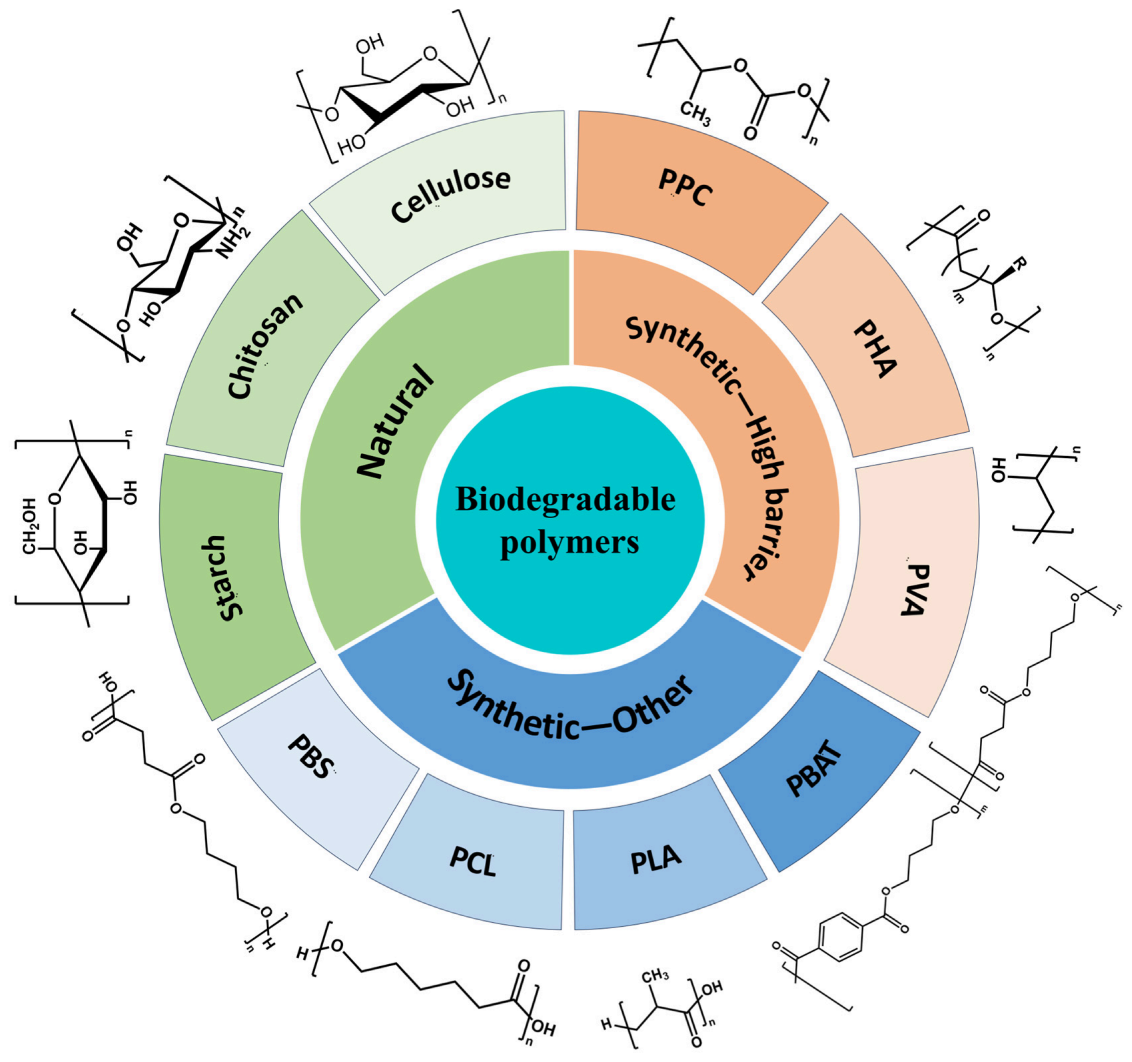
Classification and chemical structural of natural and synthetic biodegradable polymers.

Biodegradable polymers are defined as materials that can undergo full microbial-mediated degradation in natural environments (e.g., soil, seawater) or controlled systems (e.g., composting facilities, anaerobic digesters, aquaculture systems), ultimately mineralizing into CO_2_, CH_4_, H_2_O, inorganic salts, and biomass. Biodegradable polymers can be classified into natural biodegradable polymers including protein, cellulose, starch and chitosan, and synthetic biodegradable polymers including PLA, PVA, PHA, PPC, poly(butylene succinate) (PBS), PBAT, and polycaprolactone (PCL) [1,69]. Notably, PPC and PHA exhibit good water vapor and oxygen barrier properties, while PVA has an extremely outstanding oxygen barrier properties. The classification and corresponding chemical structures of natural and synthetic biodegradable polymers are shown in Figure 4.

### 3.2. Natural Biodegradable Polymers

#### 3.2.1. Protein-Based Biodegradable Polymers

Protein-based biodegradable polymers can be derived from a broad range of animal and plant origins, including animal muscles and tissues, milk, oilseeds, soybeans, wheat, corn, and other grains, each possessing distinct compositions, structures, and functions. Researchers have carried out extensive studies on the barrier, mechanical, and thermodynamic performance of protein-based packaging materials [70,71,72]. In terms of food packaging applications, packaging films manufactured from protein-based biodegradable polymers serve as edible films, such as for packaging bread and pastries. In non-food packaging sectors, polymers made from corn protein, gelatin and soy protein are used in other commercial fields, including shopping bags, agricultural films and disposable household products [73,74].

However, protein-based biodegradable polymers exhibit poorer water vapor barrier properties than conventional petroleum-based plastics, a difference that can be attributed to the hydrophilic nature of protein. In addition, protein-based polymers typically exhibit inferior mechanical properties. The barrier and mechanical performance of protein polymers can be improved by cross-linking with cross-linking agents, including glyoxal, glutaraldehyde, phenolics such as tannins, gallic acid, ferulic acid, and transglutaminase [75]. Furthermore, protein mixed with chitosan, starch and pectin can improve their functional properties, including moisture resistance and mechanical stability [76,77]. Dai et al. fabricated composite films by introducing chitosan and MMT into a gelatin matrix. The mechanical and barrier properties of gelatin films were significantly enhanced after the incorporation of MMT and chitosan surface deposition. This improvement is attributed to the rise in triple helix content and the formation of a bilayer structure, respectively [78]. Lee et al. explored the effect of different concentrations of chitosan nanoparticles (CSNPs) on the barrier and mechanical performance of chicken skin gelatin bio-nanocomposite films. The WVP value of the films containing 6% CSNPs was reduced by 52%, and the tensile strength was improved by 84% compared to that of the pure gelatin films [79].

#### 3.2.2. Cellulose-Based Biodegradable Polymers

Cellulose-based polymers have emerged as highly prospective materials for advanced packaging applications, particularly in the development of nano paper and nanocomposite films, owing to their exceptional mechanical strength, outstanding oxygen barrier properties, and superior film-forming characteristics [80,81]. However, their unique structure, containing numerous hydrophilic hydroxyl groups on the molecular chains, not only gives them excellent properties, but also brings some disadvantages, such as inferior water vapor barrier properties [82]. This limitation restricts cellulose’s effectiveness when applied to packaging materials that are sensitive to moisture. Therefore, cellulose films need to be suitably physically or chemically modified to increase their potential for application in packaging. For example, strategies include treating the surface of the cellulose substrate with biopolymers, such as whey protein, PCL, PLA, and beeswax [83,84]. Moreover, deposition of inorganic impermeable particles like mica, GO, or MMT on the surface of cellulose by the LbL method is also a common method to improve the water vapor barrier properties of cellulose [85,86,87,88].

Balasubramaniam et al. modified cellulose nanofibers (CNFs) using a range of saturated fatty acids including lauric, palmitic, and stearic acids. This modification improved the hydrophobicity of CNF without compromising its excellent mechanical performance. The experimental approach consisted of two different modification techniques: surface modification and bulk modification. Both techniques effectively increased the hydrophobicity of the films. For the CNF membrane modified by palmitic acid, the WVP decreased from 0.057 g/(s·m·Pa) to 0.007 g/(s·m·Pa) [89].

Incorporating inorganic nanosheets into cellulose membranes can enhance the barrier properties of cellulose membranes. Li et al. [85] prepared GO nanosheets/CNF nanocomposite films. Compared with pure CNF membranes, adding 1.64% of GONS to the CNF matrix reduces OP by three orders of magnitude, from 10.25 × 10^−14^ to 0.01 × 10^−14^ cm^3^ cm/(cm^−2^·s^−1^·Pa^−1^). Cellulose-based carrageenan (CR)/chitosan (CS)/MMT/CS multilayer coated composite films can be prepared by the LBL technique. Incorporating MMT into cellulose-based multilayers led to the formation of oriented “nano-brick-wall” structures on the fiber surfaces, which prolonged the diffusion path of water molecules through the matrix. The WVTR of cellulose-based composite films decreased significantly as the number of composite layers increased. When the number of composite layers reached three, the WVTR of the composite film was 418 g/(m^2^·day), which decreased by 85.6% compared with 2903 g/(m^2^·day) of the pure cellulose membrane [90]. Yue et al. prepared a transparent perhydropolysilazane-derived-SiO_x_-coated cellulose film via a simple solution dip coating followed by a UV curing technique. The cellulose—SiO_x_ film had a sandwich structure, with an OTR of 0.82 cm^3^/m^2^·day and a WVTR of 1.28 g/m^2^·day (thickness of 35 μm) [18].

#### 3.2.3. Starch-Based Biodegradable Polymers

Starch is an low-cost and broadly available polysaccharide, consisting of hundreds to thousands of glucose units linked by α-glycosidic bonds [91]. Starch-based biodegradable polymers are available from a broad range of materials, such as potato, cassava, rice, maize, and tapioca [92]. However, starch as a packaging material has inherent limitations, including poor mechanical and water vapor barrier properties, which hinder its large-scale commercial application. Furthermore, the mechanical performance of plasticized starch packaging materials tend to gradually deteriorate with time of use [93,94]. Consequently, several approaches have been put forward to enhance the barrier and mechanical performance of starch-based biodegradable polymers, including chemical modification, cross-linking, blending with various biopolymers and additives, employing different plasticizers, and developing nanocomposites [95,96,97].

Starch blended with other biodegradable polymers, like CS and cellulose, can enhance mechanical properties and gas barrier properties of starch-based materials. Jiménez-Regalado et al. [98] investigated the effect of different ratios of thermoplastic starch to chitosan (TPS:CS) on film properties. The results indicated that the protonated amino groups in chitosan increased the intermolecular forces, and thus improved the barrier properties of the starch-based films. Bacterial cellulose nanocrystal whisker/thermoplastic corn starch (BCNW/TPCS) composite films can be fabricated by the melt-blending method. The mechanical and barrier properties of the starch composite films were significantly enhanced owing to the formation of strong hydrogen bonding interactions between the filler (BCNW) and the matrix (TPCS). The WVP and OP decreased by a maximum of 46% and 95%, respectively [99].

Incorporating appropriate plasticizers into starch-based materials can effectively enhance their barrier properties [96]. Ruamcharoen et al. prepared natural rubber (NR) and cassava starch (CS) composite films. Nano-clay MMT, kaolinite (KAO) and intercalated kaolinite (DKAO) were utilized as solubilizers and plasticizers. The experimental results demonstrated that incorporating nano-clay notably enhanced the mechanical and water vapor barrier properties of NR/CS composite films. The 4% content of MMT formed a well-dispersed peel/interlayer structure in the composites, which increased the diffusion pathways for water vapor molecules. As a consequence, the WVTR of CS films and CS/NR composite films was reduced by 53% and 68%, respectively [100].

### 3.3. Synthetic Biodegradable Polymers

#### 3.3.1. High-Barrier Synthetic Biodegradable Materials

Synthetic biodegradable polymers have significant advantages in environmental protection and resource stability. However, their insufficient thermal, mechanical, and processing performance, along with inadequate oxygen and water vapor barrier properties, has prevented their widespread adoption in barrier packaging applications. Thus, extensive research has been conducted in recent years to improve the overall performance of biodegradable materials required for barrier packaging uses. Among the synthetic biodegradable polymers, PPC and PHA exhibit good water vapor and oxygen barrier properties, while PVA has the same excellent oxygen barrier properties as EVOH. The following part focuses on these high-barrier synthetic biodegradable polymers.

##### PPC-Based Biodegradable Polymers

PPC is a biodegradable polymer produced via the copolymerization of propylene oxide (PO) and CO_2_ [101]. PPC exhibits excellent transparency, barrier properties and biodegradability [102,103,104]. Since the invention of PPC by Inoue et al. in 1969, it has attracted significant attention from both academia and industry. PPC has seen widespread use in packaging materials, agricultural films, and medical supplies. As a biodegradable alternative, it shows promise in replacing traditional petroleum-derived plastics [104,105]. The main chain of PPC contains hydrophobic and polar carbonate bonds, which results in a tighter molecular arrangement and smaller free volume. Consequently, PPC exhibits a high density (1.3 g/cm^3^) and good barrier properties. The OP of pure PPC is 2.25 cm^3^·mm/(m^2^·day) at 23 °C and 0% relative humidity (RH), and the WVP is 1.05 g·mm/(m^2^·day) at 23 °C and 85% RH [106]. As a CO_2_-based polymer, PPC combines high transparency and good barrier properties, rendering it promising for packaging uses. However, PPC exhibits a lower glass transition temperature (38–42 °C) and poorer mechanical properties, including brittleness and low elongation at break (EAB), which limit its application in the field of packaging [107,108,109].

Over the past few years, to expand the application potential of PPC, researchers have extensively studied methods to improve its mechanical, thermal, and barrier properties. Table 1 summarizes the different approaches used to enhance the barrier properties of PPC and their percentage improvements. These efforts include chemical modification of PPC or the integration of inorganic particles. Zhai et al. fabricated a set of PPC/aluminum flake (ALF) composite films with varying contents of ALF by melt blending. When the ALF filler content reached 5 wt.%, the OP and WVP of the composite films were dropped by 32.4% and 75.2%, respectively, compared with those of the pure PPC films. Furthermore, the mechanical and thermal performance of the composite films were also enhanced [106]. Similarly, Li et al. used organic-modified LDH (OLDH) melt blending with PPC. With the addition of 2% OLDH, the OP and WVP of the composite film were decreased by 54% and 17%, respectively, and the tensile strength was enhanced by 83% [27].

Except for non-biodegradable inorganic particles, blending PPC with degradable polymers, such as starch, PBAT, and PLA, can enhance mechanical properties or further improve barrier properties while maintaining biodegradability of PPC [110]. Corre et al. blended polyhydroxyalkanoates (PHAs) and PPC to study their mutual contribution in barrier properties. PHA made a significant contribution to the OP and WVP of PPC. The WVP and OP of the composite film with 25% PHA added decreased by 51.5% and 46.5% (23 °C, 50%RH) respectively compared with the pure PPC film [111]. Xu et al. fabricated a three-layer biodegradable composite film consisting of PBAT/PPC/PBAT by multilayer coextrusion. The study investigated the effects of different PPC layer thicknesses, PBAT layer thicknesses and tensile speeds on the properties of the composite film. The tensile strength of the best performing composite film was increased by 200% compared to the pure PPC film, with a minimum OP of 0.821 cm^3^·mm/(cm^2^·s·bar) [112].

**Table 1:** Oxygen and water vapor barrier property enhancement of modified poly(propylene carbonate) films.

Entry	Filler	Preparation	OP cm^3^·mm/(m^2^·day)	Reduction in OP	WVP g·mm/(m^2^·day)	Reduction in WVP	References
1	OMMT	Melt blending	0.315 (23 °C, 0% RH)	44.7% at 3 wt.%	N/A	/	[113]
2	PBAT	Melt blending	0.821	/	N/A	/	[112]
3	ALF	Melt blending	0.73 (23 °C, 0% RH)	67.6% at 5 wt.%	0.79 (23 °C, 85% RH)	24.8% at 5 wt.%	[106]
4	PBAT + GO	Melt blending	N/A	/	/	83.2% at 70/1 wt.%	[114]
5	PHA	Melt blending	0.62 (23 °C, 50% RH)	46.5% at 25 wt.%	0.544 (23 °C, 50% RH)	51.5% at 25 wt.%	[111]
6	PHB + CNC	Melt blending	0.11 (23 °C, 45% RH)	94.3% at 15/1 wt.%	N/A	/	[115]
7	OLDH	Coprecipitation process	1.04 (23 °C, 0% RH)	54% at 2 wt.%	1.03 (23 °C, 85% RH)	17% at 2 wt.%	[27]
8	MMT	Mechanical mixing	0.12 (23 °C)	95.5% at 7 wt.%	N/A	/	[116]
9	GCNC	Mechanical mixing	N/A	/	23.03 (120 °C)	94.5% at 3 wt.%	[117]
10	BNNS	Mechanical mixing	1.97 (23 °C, 0% RH)	26.1% at 15 wt.%	0.649 (23 °C, 85% RH)	47.6% at 15 wt.%	[118]

Note: OMMT, organo-montmorillonite; PBAT, poly(butyleneadipate-co-terephthalate); ALF, aluminum flake; GO, graphene oxide; PHA, polyhydroxyalkanoate; PHB, polyhydroxybutyrate; CNC, cellulose nanocrystal; OLDH, organic-modified layered double hydroxides; MMT, montmorillonite; GCNC, graphitized nanocellulose; BNNS, ball-milled boron nitride.

**Figure 5:**
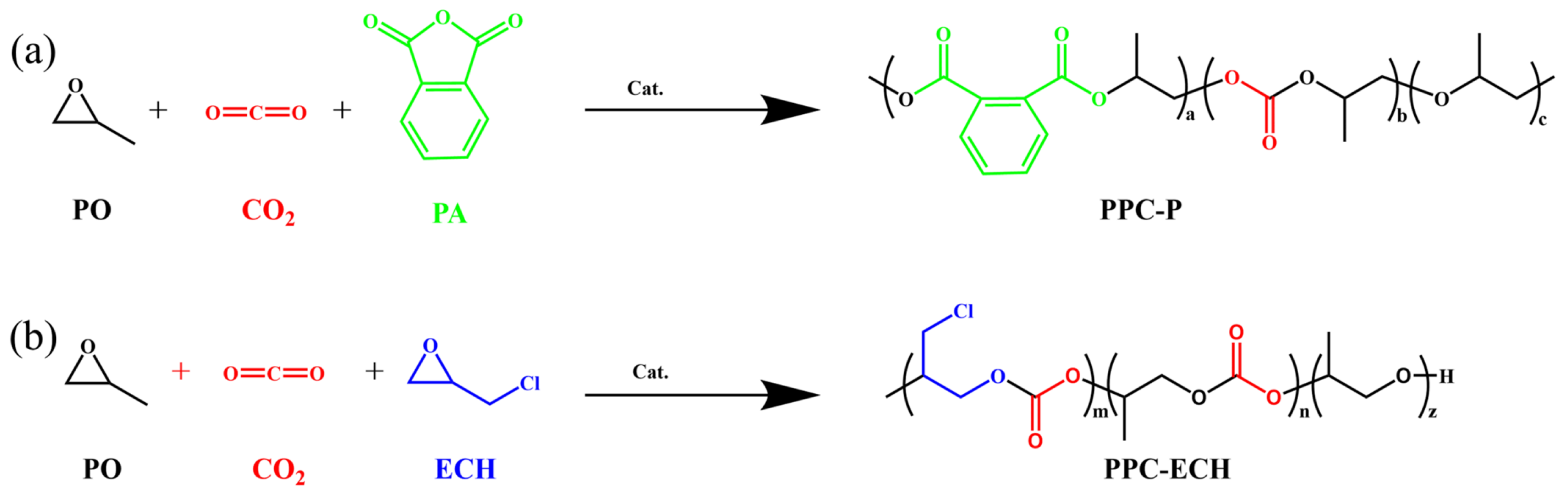
The synthesis process of (**a**) PPC-P [104] and (**b**) PPC-ECH [121].

However, the main reason hindering the large-scale application of PPC is its poor thermal performance, including a low glass transition temperature (Tg) and poor thermal stability. Therefore, PPC requires modification to enhance its thermal and mechanical properties without affecting its water vapor and oxygen barrier properties. One effective method is to introduce a third monomer in the PO/CO_2_ copolymerization. Liang et al. synthesized the terpolymer PPC-P shown in Figure 5a via copolymerization of PO, phthalic anhydride (PA) and CO_2_, using TEB as the catalyst and PPNCl as the initiator. The results demonstrate that incorporating PA with a rigid structure can effectively improve the thermal and mechanical performance of PPC. They systematically investigated the effects of feed ratios, reaction temperatures, and CO_2_ pressures on polymer characteristics. The optimal PPC-P exhibited a Tg of 47 °C, a tensile strength of 37.6 MPa, and a degradation rate similar to that of PBAT. The barrier properties was further improved, with OP and WVP reduced to 1.392 cm^3^·mm/(m^2^·day) and 0.0138 g·mm/(m^2^·day), respectively [104]. Deng et al. investigated blends of PPC-P and PBAT with varying ratios, and introduced the chain extender MDI into the blends. The 75 PPC-P/PBAT blend with 2% chain extender exhibited the best performance. The incorporation of chain extenders notably enhanced the thermal stability and EAB of PPC-P/PBAT. Subsequently, PVA and borax were coated on the PPC-P/PBAT composite film by the coating method, and the oxygen barrier properties of the coated composite film were further enhanced. The PPC-P/PBAT composite film coated with 3 wt.% borax had an OP lower than 0.0035 cm^3^·mm/(m^2^·day), reaching the lower limit detected by the instrument [119]. Yue et al. prepared the PVA/MMT nano-coatings with nacre-like microstructures, and then applied them to cellulose and PPC-P by dip-coating and flow-induced self-assembly methods. Due to the high level of dispersion and orientation of MMT nanosheets, the PVA/MMT nano-coating endows the coated substrate with significantly improved gas barrier properties. The OTR and WVTR of the cellulose/PPC-P coated membranes declined to 0.35/<0.02 cm^3^/(m^2^·day) and 13.06/0.23 g/(m^2^·day), respectively [120].

From the perspective of polymerization, using epichlorohydrin (ECH) as the third monomer to modify PPC is worthy of attention. Introducing chlorine into the main chain of PPC can increase rigidity and produce polymers with customizable performance: improved thermal stability, mechanical strength, flame retardancy, and barrier properties. Liang et al. synthesized the PPC-ECH terpolymer illustrated in Figure 5b by a one-pot-method, metal-free approach, employing a polynuclear organic boron catalyst for the tercopolymerization reaction of CO_2_, PO and ECH. The synthesized PPC-ECH-35.7, where 35.7 is the molar content of ECH, exhibits excellent barrier properties, with an OP as low as 1.31 cm^3^·mm/(m^2^·day) and a WVP as low as 0.033 g·mm/(m^2^·day) (23 °C, 85%RH). These values are notably lower than those of pure PPC or other conventional packaging materials [121]. Notably, the integration of a small quantity of ECH not only retains the biodegradability of PPC, but also significantly improves its mechanical strength, thermal stability, and flame retardancy. In addition, monomers such as epoxy-cyclohexane (CHO) [122] and phthalic anhydride (PMDA) [123] are copolymerized with CO_2_ and PO, which can also increase the Tg and mechanical performance of PPC. However, the relatively high prices of PMDA and CHO increase production costs, thereby limiting their industrial applications.

Overall, PPC exhibits excellent transparency, barrier properties, and biodegradability. However, its relatively low Tg, thermal stability, and poor mechanical properties have hindered its large-scale application. Researchers have introduced organic and inorganic substances into PPC through blending and copolymerization to increase its Tg and mechanical properties, and have achieved significant improvements.

##### PHA-Based Biodegradable Polymers

PHAs are a category of intracellular polymers produced by various bacteria, which can also be found in certain microorganisms and plants. Over the past 20 years, researchers have discovered and reported more than 150 kinds of PHAs from a wide variety of bacteria, most of which have been synthesized and investigated in the laboratory [124]. PHAs possess physical and chemical performance similar to those of synthetic plastics, including biodegradability, biocompatibility, optical activity, piezoelectricity, and gas barrier properties. PHAs have found applications in biodegradable packaging materials, tissue engineering materials and medical materials. However, the relatively high production cost of PHAs limits their large-scale application [125]. Derivatives of PHA include polyhydroxybutyrate (PHB), polyhydroxyvalerate (PHV), and copolymers of PHB and PHV (PHBV). PHB is a key type of PHA; however, its practical use is restricted by inherent brittleness and inadequate thermal stability. In order to solve the limitations of PHB, copolymers were prepared via the incorporation of 3-hydroxyvalerate or 4-hydroxybutyrate units into the PHB backbone. These copolymers are referred to as poly(3-hydroxybutyrate-co-3-hydroxyvalerate) (PHBV) and poly(3-hydroxybutyrate-co-4-hydroxybutyrate) (PHBH). These copolymers exhibit a remarkably high crystallinity of 60% and an OP below 1.3 cm^3^·mm/(m^2^·day·bar) [126]. Therefore, PHBV and PHBH have potential applications in food packaging.

Blending PHAs with other biopolymers to prepare composite films is an effective strategy for enhancing the overall performance of PHAs [1,127]. The incorporation of poly(butylene succinate-co-adipate) (PBSA) can enhance the oxygen and water vapor barrier properties of PHB [128]. Eslami et al. prepared a novel biodegradable composite film, PHBV–sandwich–PHBV composite film, where the sandwich layer consisted of thermoplastic starch (TPS), maleated TPS, or their mixture with PHBV (80/20). The composite films exhibited excellent tensile strength, water vapor and oxygen barrier properties, with tensile strengths up to 178 MPa, WVTR as low as 20.2 g/(m^2^·day), OTR beneath the detection limit of the instrument, and a degradation period of no more than six weeks [129]. Melendez-Rodriguez et al. prepared PHB-CNC-PHB composite films via the electrospinning method. The results demonstrated that the composite films exhibited favorable interlayer compatibility and enhanced mechanical and barrier properties. The 1 µm CNC interlayers reduced oxygen permeation by 86% compared to pure PHB films [130].

In addition to biopolymers, the integration of minor quantities of inorganic fillers into the PHA matrix has emerged as an effective approach to improve their mechanical and thermal performance. These inorganic fillers serve dual functions as both reinforcing and nucleating agents, providing heterogeneous nucleation sites that significantly accelerate the crystallization rate of the polymer matrix. For example, inorganic fillers such as MMT, multi-walled carbon nanotubes (MWCNTs), and boron nitride have all been demonstrated to function effectively as nucleating agents [131]. Yan et al. prepared a bio-composite (MMT/PHBH) by the solution-casting method. The results demonstrated that the incorporation of MMT elevated the crystallization rate and crystallinity of PHBH, indicating that MMT is an effective nucleating agent. The composites with 1 wt.% MMT exhibited the best thermal stability, mechanical and barrier properties. The tensile strength and modulus of elasticity of the composites were improved by 111.2% and 182.5%, respectively, compared with those of unmodified PHBH. The OTR and WVTR were decreased by 43.9% and 6.9%, respectively [132].

##### PVA-Based Biodegradable Polymers

PVA is a high-density, high-crystallinity polymer with strong adhesion. It can be processed into flexible, smooth, oil-resistant, wear-resistant and oxygen-barrier films. In addition, PVA membranes have a good affinity with the natural environment. These materials do not accumulate in the environment, nor do they cause pollution; additionally, they are non-toxic, odorless, and safe for humans [133].

PVA films are environmentally friendly functional materials that can be completely biodegraded by soil microorganisms. PVA films can be degraded to CO_2_ and water within a short timeframe, while improving the quality of the land. The molecular chain of PVA comprises a high density of hydroxyl groups. When dried, it forms a dense structure through hydrogen bonds, and its oxygen barrier properties are close to that of EVOH. However, in a humid environment, hydroxyl groups absorb water and swell, and their ability to block water vapor sharply declines [23]. If the hydroxyl groups are appropriately blocked or the diffusion path of water molecules is prolonged, the water vapor barrier properties of PVA membranes can be enhanced. Zamanian et al. prepared PVA nanocomposite membranes by the solution-casting method and added MMT and TiO_2_ nanoparticles. The WVP of PVA membranes with 1 wt.% TiO_2_ and 4 wt.% MMT decreased by 52.7%. Owing to the non-porous nature of TiO_2_ nanoparticles and MMT, an extended diffusion path results in a reduction in WVP [134]. Furthermore, the restriction of polymer chain mobility also contributes to the reduction in WVP. Wang et al. prepared PVA/MSMX/citric acid (CA) composite films. MSMX was obtained by modifying bagasse xylan with 3-mercaptopropyltrimethoxysilane (MPTMS), and CA was used as the cross-linking agent. The WVP of the PVA/MSMX/CA composite membrane was at least 1.89 g·mm/(m^2^·day), a value decreased by over two orders of magnitude compared with the unmodified PVA membrane. The excellent barrier properties of the material are ascribed to the “tortuous path effect” [135].

#### 3.3.2. Other Synthetic Biodegradable Materials

##### Poly(Lactic Acid) (PLA)-Based Biodegradable Polymers

PLA is currently the most extensively utilized biodegradable plastic in the market. PLA is mainly sourced from renewable resources, including sugar beet, corn, sugar cane, potato, cassava, wheat straw, bagasse or sawdust. In the past few years, PLA films have attracted substantial interest [136,137,138]. PLA films find widespread application in the packaging industry due to their high molecular weight, good processability, and biodegradability [25,109,139,140]. Although PLA serves as a promising biodegradable packaging material, several limitations exist in food packaging scenarios. These limitations stem from the poor thermodynamic properties, low heat distortion stability, slow crystallization rate, weak melt strength, moderate water resistance, and inadequate oxygen barrier properties of PLA. There have been extensive studies dedicated to enhancing the barrier properties of PLA.

PLA is a semi-crystalline polymer that consists of both crystalline and amorphous phases. Consequently, its barrier properties can be tuned by adjusting the supramolecular microstructure of the crystalline phase and the free volume of the amorphous phase. Bai et al. introduced nucleating agents into PLA by melt compounding, and then prepared PLA film samples via compression molding. Under the induction of the high-efficiency fibrous nucleating agent, the PLA formed a parallel-arranged “shish-kebab-like” crystalline structure with tightly interlocked internal boundaries, and the resulting dense “nano-brick-wall” structure effectively blocked the passage of gas molecules, resulting in an extremely low OP (1.718 × 10^−10^ cm^3^·mm/(m^2^·day·bar)) of the modified PLA films [141].

Numerous studies have demonstrated that the integration of nanofillers into the PLA matrix, such as nano-clay (MMT, KAO, and LDH), carbon-based nanomaterials (graphene, carbon nanotubes, or nanofibers), metal nanoparticles (silver, magnesium, titanium, and iron), inorganic nanoparticles (alumina, nano-silica, and polyhedral oligomeric silsesquioxane), or nanocellulose, can significantly enhance the barrier properties of PLA [142]. Yang et al. successfully prepared 3-aminopropyltriethoxysilane (APTES)-modified MgAl-LDH nanosheets (APTES@LDH), which were then blended with PLA by the solution method. Subsequently, the mixture was converted into biodegradable composite films with both oxygen and water vapor barrier properties using a coating technique. The OTR of the composite membrane was lower than the detection limit of 0.005 cm^3^/(m^2^·day·bar), and the WVTR was 0.026 g/(m^2^·day·bar). The improved barrier properties can be ascribed to the tortuous path effect created by 2D LDH nanosheets and the hydrogen bonding interactions between water molecules and LDH hydroxyl groups, which effectively inhibit the permeation of both oxygen and water vapor [143]. Sun et al. employed a simple flow-induced co-assembly method to fabricate highly oriented PVA/α-zirconium phosphate (ZrP) organic/inorganic hybrid nano-coatings. The PLA films were modified by the PVA/ZrP nano-coatings, and the OP was significantly reduced from 16.9 cm^3^·mm/(m^2^·day·bar) before coating modification to 0.04 cm^3^·mm/(m^2^·day·bar) and WVP was reduced from 2.14 g·mm/(m^2^·day) to 0.45 g·mm/(m^2^·day) [23]. Jung et al. fabricated a composite material by adding CNF to a PLA matrix. The tensile strength, toughness, and oxygen barrier properties of PLA were improved. The incorporation of 1 wt.% CNF enhanced the tensile strength and oxygen barrier properties of PLA by 20.3% and 47.3%, respectively [144].

##### Poly(Butylene Succinate) (PBS)-Based Biodegradable Polymers

PBS is a biodegradable polymer with oxygen/water vapor barrier properties similar to those of PLA, synthesized from succinic acid and 1,4-butanediol. PBS demonstrates outstanding thermal resistance, with heat deflection and service temperatures above 100 °C. The raw materials of PBS can be extracted from petroleum resources or obtained by fermentation from biological resources [145]. Therefore, it is one of the leading biodegradable polymers. The chemical structure, molecular weight, and crystallinity of PBS molecules have different effects on its barrier properties, mechanical and thermodynamic properties, and degradation process [146]. According to reports, the OTR of semi-crystalline PBS with a crystallinity of approximately 35% is higher than that of PLA but lower than that of PHB/PHBV and PPC [147].

The method to improve the barrier properties of PBS is generally to add a small number of inorganic materials to PBS. Inorganic materials can prolong the diffusion paths of water vapor and oxygen in PBS substrates. Di et al. prepared GO-PBS nanocomposites via in situ polymerization with diisocyanate. The even distribution of GO sheets led to a more convoluted oxygen diffusion pathway, thereby enhancing the oxygen barrier properties of the composites. Compared with pure PBS, the OP of GO-MDI-PBS decreased by 87.51% [148]. Tsou et al. doped graphene-ZnO into PBS to prepare PBS/graphene-ZnO nanocomposites. When the addition amount of graphene-ZnO is 1%wt., the WVP of the composite material decreases from 2.01 g·cm/(cm^2^·s·Pa) to 1.18 × 10^−13^ g·cm/(cm^2^·s·Pa) [149].

##### Polycaprolactone (PCL)-Based Biodegradable Polymers

PCL is a flexible and biodegradable polymer synthesized from petroleum-based ε-caprolactone monomers through ring-opening polymerization, and it is an environmentally friendly polyester. Compared with other biodegradable polymers, PCL has many advantages, including low viscosity, facile processing, favorable solvent resistance, and excellent mechanical properties [150]. However, PCL has disadvantages such as high cost and inadequate water vapor and oxygen barrier properties, which restrict the applications of PCL in high-barrier packaging [151]. Blending PCL with PPC, which has good barrier properties, is an effective approach to enhance its barrier properties without affecting its biodegradability [152].

The barrier properties of PCL can be enhanced via adding different types of nanofillers including clay, graphene, carbon nanotubes, and metal oxide nanoparticles [1]. Bujok et al. synthesized trihexyl (tetradecyl) phosphonium ionic liquid (IL)-functionalized ZnO nanoparticles (IL-ZnONPs) and layered double hydroxides (IL-LDHs), and prepared PCL/ZnONPs and PCL/LDH nanocomposites using the ring-opening polymerization (ROP) method. During the ROP process, IL-ZnONPs or IL-LDHs were uniformly dispersed in the PCL matrix, which promoted the formation of large PCL crystals. In addition, the surface-bound ILs acted as nanoscale surfactants, compatibilizers, and thermal stabilizers in the composites. As a result, PCL-based composites exhibited better mechanical, thermal stability and oxygen/water vapor barrier properties [153].

Tribehenin glycerol esters (C18) exhibit excellent gas barrier properties attributed to their hydrophobic nature and tightly packed crystalline structure. Therefore, they have been employed as additives to improve the barrier properties of PCL. Ding et al. [154] fabricated poly(ε-caprolactone)/C18 (PCL/C18) composites by the multi-step biaxial stretching and extrusion method. C18 enhances the hydrophobicity of the material and increased the diffusion pathway of gas molecules, thus improving the oxygen/water vapor barrier properties of the composite. With the addition of 30% C18, the water contact angle on the material surface increased from 79° to 132°, and the WVP and the OP were decreased by 83.9% and 39.2%, respectively.

##### PBAT-Based Biodegradable Polymers

PBAT is a copolymer consisting of butylene glycol adipate and butylene glycol terephthalate. Although PBAT is sourced from petroleum resources, it is a biodegradable synthetic polymer material. PBAT has found use across a broad range of applications, including plastic films, packaging materials, and soil mulches due to its similar properties to PE, such as good flexibility, ductility, and heat resistance [155]. However, the poor gas barrier properties of pure PBAT restrict its use as a barrier packaging material.

The barrier properties of PBAT can be enhanced via various methods, and blending or multilayer coextrusion with other economical biodegradable polymers is one effective method. PPC is a biodegradable polymer with high gas barrier properties and lower cost than PBAT. However, PPC has poor mechanical properties. Therefore, blending PPC with PBAT can prepare polymers with excellent mechanical, thermal, and barrier properties, thus broadening their potential applications in biodegradable packaging materials. Jiang et al. prepared PPC/PBAT blends with 30% to 50% PBAT content by the melt-blending method. The prepared blends had excellent mechanical and gas barrier properties [156].

To fully utilize the performance advantages of each component in PBAT/PPC blends, interfacial modification is required during blending to improve compatibility. Yang et al. fabricated bio-composites composed of PBAT, PPC and epoxy chain extender ADR 4468 using a torque rheometer and the melt-mixing technique. The study investigated the impact of different feed ratios on the barrier properties of the composite materials, and the WVTR of the composites decreased by 50% when the contents of PPC and ADR 4468 were 30% and 3%, respectively. Meanwhile, the tensile strength and EAB improved from 19.5 MPa and 1184% to 26.9 MPa and 1443%, respectively [30]. Xie et al. prepared PBAT/PPC composite films via melt blending and the film blowing technique, with triethylene glycol isocyanurate (TGIC) as compatibilizer. The experimental results showed that the addition of TGIC could effectively improve the interfacial compatibility between PBAT and PPC and enhance the thermal stability of the composites. The effect of the content of TGIC on the mechanical and barrier properties of the composite films was also investigated. After adding 1 wt.% TGIC to PBAT/PPC, the tensile strength of the composite film was increased from 22.7 MPa to 30.8 MPa, the EAB was improved from 605% to 860%, and the WVTR was reduced from 1.97 g·mm/(m^2^·day) to 1.32 g·mm/(m^2^·day) [29].

The barrier performance of modified biodegradable materials is highly dependent on the modification strategy and the resulting structural evolution. Table 2 summarizes representative research results enabling horizontal comparison between pristine and modified films. Taking PBS, PCL, and PBAT/PPC as typical examples, filler dispersion state, interfacial interactions, and phase morphology jointly determine the free volume and gas diffusion pathways, and thus govern the ultimate oxygen and water vapor barrier properties.

## 4. Conclusions and Perspectives

Since the 1950s, the accumulation of plastic waste has caused increasing environmental pollution, especially plastic packaging, which in particular accounts for about 50% of plastic waste. Biodegradable polymers serve as an outstanding alternative to traditional petroleum-based plastic packaging, but their oxygen and water vapor barrier properties and mechanical properties still need to be improved. In the past decades, researchers have enhanced the barrier and mechanical properties of biodegradable polymer films through surface coating, multilayer coextrusion, and multilayer lamination. However, their performance still lags behind that of traditional petroleum-based plastic films.

This paper provides an overview of commonly used biodegradable plastics, including natural biomass polymers (protein, cellulose, chitosan, and starch) and synthetic polymers (PLA, PBS, PBAT, PHB, PPC, PHA, PCL, and PVA), with a focus on the gas/water vapor barrier properties. The influence of the structure and processing of biodegradable polymers on the gas/water vapor barrier properties is summarized, along with advancements in research on improving barrier and mechanical properties through various methods, including surface coating, multilayer compositing, blending, copolymerization and modification, and nanocomposites and nanotechnology.

Despite the progress in the above technologies to improve the oxygen/water vapor barrier of biodegradable plastics, challenges remain in reducing costs and developing new technologies. Some potential directions and prospects include:

(1) Reducing the cost of biodegradable polymer packaging materials.
(2) Improving the degradation conditions of polymers by reducing the addition of non-degradable compounds and increasing their biodegradability.
(3) Improving the overall performance of polymer materials. Many of the modification methods mentioned above either improve only the barrier properties, only the mechanical properties, or both the barrier and mechanical properties, but with a concomitant increase in manufacturing costs.
(4) Optimizing life cycle assessment (LCA) to balance barrier properties and degradation efficiency.
(5) CO_2_ permeability, UV shielding, nanofiller/cross-linker toxicity, substance migration and real-environmental biodegradation are vital food packaging-related metrics beyond this review’s oxygen/water vapor barrier focus, which deserve systematic elaboration in independent follow-up reviews.

## Figures and Tables

**Table 2:** Barrier property enhancement of modified biodegradable polymer films via various strategies.

Entry	Polymer Matrix	Modification Strategy	Key Structural Evolution	Reduction in WVP	Reduction in OP	References
1	PLA	APTES-grafted LDH blending	Oriented LDH hydrogen-bond network	94.1% at 10 wt.%	>99% at 10 wt.%	[143]
2		Oriented ZrP PVA coating	Parallel nanosheet brick-wall structure	78.9% at 20 wt.%	99.7% at 20 wt.%	[23]
3		TEC-assisted CNF melt blending	Uniform CNF network formation	78.6% at 1 wt.%	47.3% at 1 wt.%	[144]
4	PBS	MDI-bridged GO in situ polymerization	Covalent intercalated GO uniform dispersion	42.0 at 0.5 wt.%	87.5% at 0.5 wt.%	[148]
5		Graphene-ZnO melt compounding	Coordination-bonded filler network	42.6 at 0.2 wt.%	/	[149]
6	PCL	IL-modified LDH in situ ROP	Exfoliated uniform LDH dispersion	10.0% at 1 wt.%	12.0% at 1 wt.%	[153]
7		Glycerol tristearate melt blending	Hydrophobic dispersed microdomains	81.4% at 30 wt.%	39.2% at 30 wt.%	[154]
8	PBAT/ PPC	TGIC reactive melt blending	Finer dispersed	33.0% at 1 wt.%	/	[29]
9		ADR chain extender blending	Uniform phase dispersion	50.0% at 3 wt.%	/	[30]
10		EMA-GMA reactive compatibilization	Minimized dispersed droplets	43.7% at 1 wt.%	/	[156]

Note: APTES, 3-aminopropyltriethoxysilane; LDH, layered double hydroxide; TEC, triethyl citrate; CNF, cellulose nanofiber; MDI, 4,4′-diphenylmethane diisocyanate; GO, grapheneoxide; IL, ionic liquid; ROP, ring-opening polymerization; TGIC, triglycidyl isocyanurate; EMA-GMA, ethylene-methyl acrylate–glycidyl methacrylate.

## Data Availability

No new data were created or analyzed in this study. Data sharing is not applicable to this article.
